# Supplementary material for: Modeling Multiscale Architecture of Biofilm Extracellular Matrix and Its Role in Oxygen Transport
Source: Biotechnol Bioeng. 2026 Jul 5;123(10):2758–70. doi: 10.1002/bit.70295 (PMC13576833; doi:10.1002/bit.70295)
Supplement: Supplementary file 1 [file BIT-123-2758-s001.docx]

**Modelling multiscale architecture of biofilm extracellular matrix and its role in oxygen transport**

Raghu K. Moorthy^1^, Eoin Casey^1,*^

^1^School of Chemical and Bioprocess Engineering, University College Dublin, Belfield, Dublin, Ireland

**Figure S1:** Schematic diagram of the typical distribution trends observed with count of cell-capsule structures in a given biofilm morphology (left panel) and the corresponding mathematical transformation to the density function (right panel).

**Figure S2**: Schematic diagram of the control volume for description of cell-capsule patterns and subsequent re-scale using biofilm thickness to constitute the biofilm phase in the multiscale model.

**Figure S3**: Schematic diagram of the numerical implementation for the multiscale model.

**Figure S4**: Scatter plot shows the decrease in effective diffusion coefficient of oxygen in the capsule theoretically estimated using experimental data on Young’s modulus.

**Table S1**: Different rheological case studies of bacterial capsule from previous literature.

**Table S2**: Different rheological case studies of biofilm structure from previous literature.

**Table S3**: Theoretical estimates of diffusion coefficient of oxygen in capsule.

**Table S4**: Theoretical estimates of diffusion coefficient of oxygen in biofilm.

**Figure S1**: Schematic diagram of the typical distribution trends observed with count of cell-capsule structures in a given biofilm morphology (left panel) and the corresponding mathematical transformation to the density function (right panel). Note: Dimensions are not to scale.

**Figure S2**: Schematic diagram of the control volume for description of cell-capsule patterns and subsequent re-scale using biofilm thickness to constitute the biofilm phase in the multiscale model. Note: d_core_ is cell diameter, L_p_ is capsule thickness, x and z represent radial and lateral directions respectively. Dimensions are not to scale.

**Figure S3**: Schematic diagram of the numerical implementation for the multiscale model.

**Figure S4**: Scatter plot shows the decrease in effective diffusion coefficient of oxygen in the capsule theoretically estimated using experimental data on Young’s modulus. The green-coloured symbols refer to the rheological studies on capsule, and the blue-coloured symbols refer to the rheological studies on biofilms. Data points are collected from limited experimental studies available from previous literature. Note: Both coordinate axes are plotted in logarithmic scale for better visualization. Error bars represent standard deviation on account of the curve fitting exercise using a set of empirical models [6].

**Theoretical estimates of diffusion coefficient of oxygen using elastic deformation of biofilm matrix**

Using a facile method from poreelastic theory presented by Hu and co-workers [6], it is possible to characterize soft materials using their elastic behavior. Recorded curves of measured force upon indentation of the material with time are obtained, when a certain type of indenter is pressed into the material to a fixed depth. The above elastic behavior is a function of the indenter type used in the atomic force microscopy (AFM) studies (spherical or conical type is commonly used for biofilm material, see references cited for more details, Tables S1 and S2).

For the experimentally measured force (in nN) normalized by the depth of indentation, F(t), the numerical data is extracted using a plotting software [13] from the above AFM studies focusing either on the capsule or the biofilm material. The theoretical expressions for a spherical and a conical indenter is given by Equations S1 and S2 respectively:

Spherical:

(S1)

Conical:

(S2)

where, D_eff_ (in m^2^ s^-1^) is the effective diffusion coefficient of the mobile molecules (oxygen in the present study), normalized time is $X= \frac{t}{a\text{2}}$,

for a given *t* = analysis time (in seconds), and *a* = contact size of the indenter (in nm for the capsule, or in µm for the biofilm material).

Above theoretical measured force curves are fitted with different values of diffusion coefficient of oxygen, using an initial guess value of 1*10^-11^ m^2^ s^-1^. Upon overlap with the experimentally measured force curves for which corresponding elastic modulus or Young’s modulus (in kPa) is known, it is determined as the best fit for the estimation of the effective diffusion coefficient (with upper and lower limits at 95% confidence interval, by using ‘Nonlinear curve fit’ analysis) (Prism 10, GraphPad Software LLC, USA). The above value is then compared with the previous literature on biofilm material (see Table S2) to obtain the conversion factors as 86400*10^-7^ (for capsule) and 86400*10^-6^ (for biofilm) respectively and to report in the dimensional units of m^2^ day^-1^. In our present study, these values are also compared against an effective diffusion coefficient of oxygen as 5*10^-4^ in the bulk phase (D_bulk_) of the biofilm system to determine a diffusivity ratio (as percentage difference). On an average, it is calculated as 90% for the capsule and 60% for the biofilm when compared to that in the bulk phase (more sample calculation details are provided in the supplementary worksheet at our GitHub project repository webpage: <https://github.com/raghukrm/ERC-ABSOLUTE-Biofilm-Models-BPM/>). In our theoretical investigations, we thus considered a conservative estimate of effective diffusion coefficient of 0.2 times D_bulk_ for the capsule when compared to oxygen diffusion in the bulk phase (that is, D_s_ = 0.2*D_bulk_).

**Table S1**: Different rheological case studies of bacterial capsule from previous literature

| Capsule thickness (µm) | Young's modulus (kPa) | ^Diffusion coefficient, D_s_ (m^2^ day^-1^) | Reference |
| --- | --- | --- | --- |
| 0.7 | NA | NA | [[1]](https://doi.org/10.1128/aem.45.4.1188-1195.1983) |
| 0.42 | 6 | 5.2*10^-5^ | [[2]](https://doi.org/10.1039/C3SM51325D) |
| 0.27 | 320 | 4.4*10^-5^ | [[3]](https://doi.org/10.1021/acsnano.9b08631) |
| 0.03 | 140 | 4.2*10^-5^ | [[4]](https://doi.org/10.1016/j.resmic.2021.103865) |
| 0.35 | NA | NA | [[5]](https://doi.org/10.1128/spectrum.03087-24) |
| Typical capsule value of Young’s modulus (kPa) | | | 155.3 (mean) |

^Estimated from empirical models for atomic force microscopy (AFM) indenters reported by Hu and co-workers [6]

NA – Information on AFM studies are not available

**Table S2**: Different rheological case studies of biofilm structure^a^ from previous literature

| Biofilm thickness (µm) | Young's modulus (kPa) | ^Diffusion coefficient, D_s_ (m^2^ day^-1^) | Reference |
| --- | --- | --- | --- |
| 20 | 2.10 | NA | [7] |
| 44 | 2.00 | 1.6*10^-4^ | [8] |
| 20 | 0.10 | 2.4*10^-4^ | [9] |
| 154.5 | 0.75 | 1.6*10^-4^ | [10] |
| 500 | 0.70 | NA | [11] |
| Typical biofilms value of Young’s modulus (kPa) | | | 1.2 (mean) |

^a^Cells + EPS matrix

^Estimated from empirical models for atomic force microscopy (AFM) indenters reported by Hu and co-workers [6]

NA – Information on AFM studies are not available

**Table S3**: Theoretical estimates of diffusion coefficient of oxygen in capsule (^D_s_ = 0.1 D_bulk_)

| Minimum D_s_ (m^2^ day^-1^) | Maximum D_s_ (m^2^ day^-1^) | Standard error in D_s_ (m^2^ day^-1^) | Reference |
| --- | --- | --- | --- |
| NA | NA | NA | [[1]](https://doi.org/10.1128/aem.45.4.1188-1195.1983) |
| 3.9*10^-5^ | 7.0*10^-5^ | 15.6*10^-6^ | [[2]](https://doi.org/10.1039/C3SM51325D) |
| 3.6*10^-5^ | 5.4*10^-5^ | 9.0*10^-6^ | [[3]](https://doi.org/10.1021/acsnano.9b08631) |
| 3.7*10^-5^ | 4.8*10^-5^ | 5.2*10^-6^ | [[4]](https://doi.org/10.1016/j.resmic.2021.103865) |
| NA | NA | NA | [[5]](https://doi.org/10.1128/spectrum.03087-24) |
| ^#^Typical value of D_s_ / D_bulk_ | | | 0.1 (mean) |

^Estimated from empirical models for atomic force microscopy (AFM) indenters reported by Hu and co-workers [6]

^#^Estimated by using diffusion coefficient of oxygen in bulk phase (D_bulk_) as 5*10^-4^ m^2^ day^-1^

NA – Information on AFM studies are not available

**Table S4**: Theoretical estimates of diffusion coefficient of oxygen in biofilm (^D = 0.4 D_bulk_)

| Minimum D (m^2^ day^-1^) | Maximum D (m^2^ day^-1^) | Standard error in D (m^2^ day^-1^) | Reference |
| --- | --- | --- | --- |
| NA | NA | NA | [7] |
| 1.3*10^-4^ | 2.0*10^-4^ | 3.6*10^-5^ | [8] |
| 1.6*10^-4^ | 3.8*10^-4^ | 10.6*10^-5^ | [9] |
| 1.0*10^-4^ | 1.9*10^-4^ | 4.9*10^-5^ | [10] |
| NA | NA | NA | [11] |
| ^#^Typical value of D / D_bulk_ | | | 0.4 (mean) |

^Estimated from empirical models for atomic force microscopy (AFM) indenters reported by Hu and co-workers [6]

^#^Estimated by using diffusion coefficient of oxygen in bulk phase (D_bulk_) as 5*10^-4^ m^2^ day^-1^

NA – Information on AFM studies are not available

**References**:

[1] L. R. Bakken, R. A. Olsen, Buoyant densities and dry-matter contents of microorganisms: Conversion of a measured biovolume into biomass, *Appl. Environ. Microbiol.* 45 (1983) 1188–1195. doi:10.1128/aem.45.4.1188-1195.1983.

[2] H. Wang, J. J. Wilksch, T. Lithgow, R. A. Strugnell, M. L. Gee, Nanomechanics measurements of live bacteria reveal a mechanism for bacterial cell protection: the polysaccharide capsule in klebsiella is a responsive polymer hydrogel that adapts to osmotic stress, *Soft Matter* 9 (2013) 7560. doi:10.1039/c3sm51325d.

[3] H. Marshall, S. Aguayo, M. Kilian, F. Petersen, L. Bozec, J. Brown, In vivo relationship between the nano-biomechanical properties of streptococcal polysaccharide capsules and virulence phenotype, *ACS Nano* 14 (2020) 1070–1083. doi:10.1021/acsnano.9b08631.

[4] D. E. Manno, A. Tala, M. Calcagnile, S. C. Resta, P. Alifano, A. Serra, Surface architecture of neisseria meningitidis capsule and outer membrane as revealed by atomic force microscopy, *Res. Microbiol.* 172 (2021) 103865. doi:10.1016/j.resmic.2021.103865.

[5] H. Eichner, C. Wu, M. Cammer, E. N. H. Tran, T. R. Hirst, J. C. Paton, J. N. Weiser, Intra-serotype variation of streptococcus pneumoniae capsule and its quantification, *Microbiol. Spectr.* 13 (4 2025). doi:10.1128/spectrum.03087-24.

[6] Y. Hu, X. Zhao, J. J. Vlassak, Z. Suo, Using indentation to characterize the poroelasticity of gels, *Appl. Phys. Lett.* 96 (2010). doi:10.1063/1.3370354.

[7] A. P. Mosier, A. E. Kaloyeros, N. C. Cady, A novel microfluidic device for the in situ optical and mechanical analysis of bacterial biofilms, *J. Microbiol. Methods* 91 (2012) 198–204. doi:10.1016/j.mimet.2012.07.006.

[8] A. Safari, O. Habimana, A. Allen, E. Casey, The significance of calcium ions on pseudomonas fluorescens biofilms – a structural and mechanical study, *Biofouling* 30 (2014) 859–869. doi: 10.1080/08927014.2014.938648.

[9] G. Zeng, B. S. Vad, M. S. Dueholm, G. Christiansen, M. Nilsson, T. Tolker-Nielsen, P. H. Nielsen, R. L. Meyer, D. E. Otzen, Functional bacterial amyloid increases pseudomonas biofilm hydrophobicity and stiffness, *Front. Microbiol.* 6 (2015). doi:10.3389/fmicb.2015.01099

[10] M. I. Hasan, S. Aggarwal, Matrix matters: How extracellular substances shape biofilm structure and mechanical properties, *Colloids Surf., B* 246 (2025) 114341. doi:10.1016/j.colsurfb.2024.114341.

[11] C. Picioreanu, F. Blauert, H. Horn, M. Wagner, Determination of mechanical properties of biofilms by modelling the deformation measured using optical coherence tomography, *Water Res.* 145 (2018) 588–598. doi:10.1016/j.watres.2018.08.070.

[13] WebPlotDigitizer (WPD), Version 3.4 (beta) or 5.2, https://automeris.io (accessed online on 29 September 2025). GitHub webpage: https://github.com/automeris-io/WebPlotDigitizer
